# Fungal infection and aflatoxin contamination in maize collected from Gedeo zone, Ethiopia

**DOI:** 10.1186/s40064-016-2485-x

**Published:** 2016-06-17

**Authors:** Nitin M. Chauhan, Alemayehu P. Washe, Tesfaye Minota

**Affiliations:** College of Natural and Computational Sciences, Dilla University, P.O. Box 419, Dilla, Ethiopia

**Keywords:** Mycotoxins, Biosensor, Cancer, *Aspergillus*, Thin layer chromatography

## Abstract

Aflatoxins contamination of maize exhibits a serious threat to human and animal health over the past few decades. To protect the safety of food commodities, regular monitoring for afltoxins in food is necessary. In the proposed study, we have followed a rapid and sensitive biosensor approach as well as thin layer chromatography method for quantification of aflatoxins. Our data demonstrate that all the samples tested were beyond the safety level of aflatoxins as determined by Food and Drug Administration and European Union. Results of fungal mycoflora evidenced the massive presence of *Aspergillus* species (75 %) followed by *Fusarium* (11 %), *Penicillium* (8 %) and *Trichoderma* (6 %) as characterized by biochemical and sporulation properties. Use of internationally developed biosensor for detection of fungal toxin in this work is the first approach that was utilized in the developing country like Ethiopia. In the end, we conclude that fungal contaminant and there metabolites are potential threat to the agricultural industry and require urgent intervention.

## Background

Mycotoxins i.e. aflatoxins represents the class of fungal polyketide secondary metabolites which are mainly produced by two fungi viz. *Aspergillus flavus* and *Aspergillus parasiticus* (Bennett and Klich 2003). Both the fungi are reported to produce four principle kinds of aflatoxins i.e. aflatoxin B_1_ (AFB_1_), aflatoxin B_2_ (AFB_2_), aflatoxin G_1_ (AFG_1_) and aflatoxin G_2_ (AFG_2_). Among these four classes of aflatoxins, AFB_1_ is predominant in nature and functionally carcinogenic in animal models if the toxicity exceeds threshold level (CAST 2003; Bennett and Klich 2003). The agricultural commodities that are prone to aflatoxins toxicity are corn and corn products, peanuts, cottonseed, milo, animal feed and majority of tree nuts (Beatriz et al. 2005; Binder et al. 2007). Aflatoxins toxicity has always remained a topic of debate in terms of international market as well as economic development of country which are part of trade market. To overcome these challenges many countries have set maximum acceptable levels of aflatoxins in food and food products and animal feed (Diener et al. 1987; European Commission 2006).

Previous studies proposed that the occurrence of aflatoxins in food products mainly influenced by favorable conditions such as high moisture content and temperature (Wu et al. 2011). The extent of contamination by aflatoxins also varies with different geographic location, agricultural and agronomic practices, storage condition of crops and more importantly processing of food materials under favorable temperature and humidity conditions (Chauhan et al. 2008). In many developing countries of Africa continent, aflatoxins toxicity of food have been companion with increase risk of hepatocellular carcinoma in the presence of hepatitis B virus infection (Henry et al. 1999) and esophageal cancer respectively (Wild and Turner 2002). Intensive exposures of AFB1 at a concentration in excess of 2 ppm are reported to cause non-specific liver problems and death within few days. Whereas, chronic effect of AFB_1_ leads to immunosuppression and nutritional deficiency (Peraica et al. 1999).

Maize as an agricultural commodity is considered as one of the best substrate for the fungi to grow and produce toxicogenesis. Many surveys across the globe showed that this crop can be highly contaminated with aflatoxins (Munkvold 2003). Ethiopia crop agriculture is very complicated, involving substantial alteration in crops cultivated around the different parts of country (Chilot et al. 1998). The maize crop is third most important crop in Ethiopia after wheat and teff and accounts for largest share in total crop production (Befekadu and Berhanu 2000). Production of maize sharply expanded from 2.5 million tons in 2003–2004 to 8 million tons in 2012–2013 (Bonger et al. 2004). Maximum quantities of maize produced are stored under poor and unsatisfactory storage conditions for considerable period of time. Traditional storage of maize in Ethiopia is like made up of mud, bamboo strips, and pits. In comparison of these storage conditions, recent technology involves storage of maize in polyethylene bags and gunny bags (Anjum et al. 2012). Previous reports proposed that extended storage of maize under unacceptable storage conditions enhances fungal growth which promotes the production of respective mycotoxins (Chauhan et al. 2008).

Despite the fact that maize is a crucial food to Ethiopian population and is vulnerable to mycotoxins risk due to different geographical and climatic conditions and poor handling of crop and storage, limited surveys have been reported on the relation of fungal mycotoxins in the crop and ways to protect the food from contamination in Ethiopia (Alemu 2008). Therefore the aim of the proposed work is to determine the fungal load of maize sample from Dilla region of Ethiopia and quantify the concentration of aflatoxins by using rapid and sensitive technique. In the present study, we used an immunochromatographic assay and thin layer chromatography assay for quantification of aflatoxin in maize samples. Thus, use of internationally developed biosensor for detection of aflatoxins in this work is the first approach that was developed in the developing countries like Ethiopia and results are discussed below.

## Methods

### Materials

Reveal Q+ aflatoxin test kit (Lot No. 203322, Neogen Corporation, USA) was used for quantitative analysis of aflatoxins in maize. Mycotoxin biosensor was purchased from Mobile Assay Inc., 150 Murray Street, PO Box 96, Nowot with Wireless Nexus 7 inch Tablet inbuilt with Android 4.0 operating system, GPS tracking and mReader Software for measuring the intensity of band developed on Reveal Q+ Aflatoxin Test Strips (Neogen Corporation, USA). The assay is based on single-step lateral flow immunocharomatographic principle with competitive immunoassay format (Mobile Assay Inc; Neogen Corporation, USA).

### Analytical standard chemicals

Different standards of aflatoxins were obtained from Hi-Media Laboratories Ltd. Mumbai, India. Preparation of standard solution was done by referring to the Manual of Official Methods of Analysis of Association of Official Analytical Chemists (AOAC 1995). From the stock solutions of each toxin as determined by UV-Spectrophotometer (UV-1800, Shimadzu, Japan), a working standard of 25, 50, 75, 100, 125, 150, and 200 ppb for AFB_1_, AFB_2_, AFB_3_ and AFB_4_ was prepared in benzene: acetonitrile (98:2 v/v) solution. All the media components and chemicals were purchased from Hi-Media Laboratories Ltd. Mumbai, India.

### Study site

The study was carried out in Dilla town of Gedeo zone located in South Nations Nationalities and Peoples Region (SNNPR) of south Ethiopia. The place is located at 86 km from regional capital Hawassa and 359 km from nation capital Addis Ababa. Five different Gedeo zones namely Dilla Zuria, Yirgachaffe, Kochere, Qisha and Wonago were visited for collection of maize samples.

### Sampling

A total number of 150 different maize samples were collected from different Gedeo zones as stated above. All the samples were randomly selected from local markets, store house, flour mills, grain retailers and street corn fruit seller. Commodities samples included dry maize flour, freshly harvested corn fruits and dry maize kernels (Table 1).

**Table 1:** Distribution of maize samples on the basis of location and types

Sample matrix	Sample location	Sample code	Number of samples	Percentage of samples from total samples
Dry maize flour	Dilla Zuria	MS1-MS26	26	39
	Yirgachaffe	MS56-MS70	15	23
	Kochere	MS86-MS98	13	20
	Qisha	MS107-MS113	7	11
	Wonago	MS136-MS139	4	6
Freshly harvested corn fruit	Dilla Zuria	MS27-MS47	21	32
	Yirgachaffe	MS71-MS79	9	14
	Kochere	MS99-MS104	6	9
	Qisha	MS114-MS124	11	17
	Wonago	MS140-MS146	7	11
Dry maize kernels	Dilla Zuria	MS48-MS55	8	12
	Yirgachaffe	MS80-MS85	6	9
	Kochere	MS105-MS106	2	3
	Qisha	MS125-MS135	11	17
	Wonago	MS147-MS150	4	6

All the samples were randomly selected from local markets, store house, flour mills, grain retailers and street corn fruit seller from different location as shown in table

### Sample preparation and aflatoxin quantification

#### Aflatoxin quantification by using biosensor based immunochromatographic assay

The aflatoxins were extracted as per manufactures protocol (Mobile Assay Inc; Neogen Corporation, USA). Briefly, different samples were bring to laboratory and grind or paste so at least 75 % of material passes through a 20 mesh sieve, about the particle size of fine instant coffee. Aflatoxins were extracted by mixing 1 part of sample to 5 parts of 65 % ethanol (HPLC grade, HI-Media Laboratories Ltd. Mumbai, India) and were vigorously vortex for 3 min. The samples were allowed to settled and then filter with syringe filter and finally utilized for quantification of aflatoxins by using Reveal Q+ aflatoxin test strip (Neogen Corporation, USA).

#### Quantification of aflatoxin by using thin layer chromatography

Quantification of aflatoxin was done according to the methodology described previously (Soares and Rodriguez-Amaya 1989) by using thin layer chromatography. Briefly, 50 grams of sample were homogenized in a blender containing a solution mixture of 270 ml of methanol and 30 ml of potassium chloride for 5 min. The mixture was filtered using Whatmann filter paper. 150 ml of filtrate was transferred to a glass containing a solution mixture of 150 ml of 30 % ammonium sulfate and 50 ml of Celite. Again the mixture was filter using Whatmann filter paper. 150 ml of filtrate was transferred to a separating funnel and was filled with 150 ml of water and twice partitioned with 10 ml of chloroform. 5 ml of solution from both the chloroform partition were combined. The mixture was evaporated in a water bath at 80 °C. The extract was spotted along with working standards with the use of Autospotter on TLC plate (Silica Gel 60G, Merck). The plate was developed in an unsaturated tank containing toluene–ethyl acetate–chloroform–formic acid (70:50:50:20, v/v). The aflatoxins were visualized by the incidence of UV light. For quantification of afltoxin, known volume of samples and standards were applied to TLC plate. The plates were developed as described above in the respective solvent. All calculations were done according to the manual of to the Manual of Official Methods of Analysis of AOAC (AOAC 1995). The identity of aflatoxins was also confirmed by reaction with its derivatives i.e. trifluoroacetic acid according to Przybykski (1975).

### Determination of fungal species and population

To detect the presence of fungi in maize samples fungal bioassay was done. Briefly, twenty gram of each sample was dissolved in 180 ml of sterile saline solution. One ml of above solution was aseptically spread on Potato Dextrose Agar (Hi-Media Laboratories Ltd. Mumbai, India) and plates were incubated at 30 °C for 7 days and After incubation they were identified to genus and species level according to taxonomic keys and guides available for the kingdom fungi (Pitt and Hocking 2009).

### Statistical analysis

The differences in aflatoxins concentration in maize between the Gedeo zones, Ethiopia were compared by ANOVA in PAST 3.11 software (Hammer et al. 2001). *P* < 0.05 was considered statistically significant.

## Results

### Aflatoxins contamination of maize samples

All the maize samples intended for human consumption tested by us shown aflatoxins toxicity higher than those recommended by Food and Drug Administration (FDA) and European Union (EU) regulatory levels as determined by immunochromatographic assay and thin layer chromatography. Results of immunochromatographic assay reveal that mean aflatoxins concentration for all samples was observed as 53 ppb. Out of total 150 numbers of samples, 53 % (80 samples) possesses more than 50 ppb concentration of aflatoxins while, 38 % (57 samples) have the aflatoxins level of 40–50 ppb. In the remaining 9 % (13 samples), aflatoxins concentration was found to be in the range of 20–40 ppb (Table 2). Whereas, results of thin layer chromatography demonstrated 52.1 ppb as a mean aflatoxin concentration for all 150 samples tested. Among 150 samples tested, 56 % (84 samples) possesses aflatoxin concentration more than 50 ppb. While, 28 % (42 samples) showed aflatoxin concentration in the range of 40–50 ppb and 16 % (24 samples) has aflatoxin concentration in the range of 20–40 ppb (Table 3). There was no significant differences were observed in the different maize commodities as well as no correlation with different locality devoid of the two different methodologies used for quantification of aflatoxin in this study (*P* = 0.567). Average aflatoxins concentration for dry maize flour, corn fruit and dry maize seeds resulted in 53.89, 52.47 and 49.79 ppb respectively as determined by immunochroatographic assay (Table 2). While mean concentrations of aflatoxins for dry maize flour, corn fruit and dry maize seeds were found to be 54.86, 50.87 and 48.29 ppb as determined by thin layer chromatography (Table 3).

**Table 2:** Concentrations of aflatoxins contaminated maize samples as determined by immunochromatographic assay

Sample no.	Concentration of aflatoxins (ppb)	Sample no.	Concentration of aflatoxins (ppb)	Sample no.	Concentration of aflatoxins (ppb)
MS1	40.2	MS26	46.8	MS51	34.23
MS2	38.6	MS27	50.67	MS52	44.08
MS3	40.71	MS28	33.49	MS53	43.28
MS4	43.21	MS29	47.07	MS54	43.59
MS5	43.73	MS30	51.78	MS55	64.7
MS6	39.94	MS31	55.26	MS56	50.29
MS7	57.7	MS32	60.29	MS57	41.86
MS8	56.9	MS33	49.53	MS58	51.62
MS9	43.38	MS34	48.13	MS59	80.98
MS10	47.35	MS35	54.45	MS60	79.26
MS11	50.23	MS36	63	MS61	77.67
MS12	43.48	MS37	43.9	MS62	88.47
MS13	45.38	MS38	43.77	MS63	66.5
MS14	32.34	MS39	50.48	MS64	83.23
MS15	74.09	MS40	43.04	MS65	53.77
MS16	47.11	MS41	38.45	MS66	66.55
MS17	52.25	MS42	41.8	MS67	49.19
MS18	53.12	MS43	61.24	MS68	44.14
MS19	47.4	MS44	38.14	MS69	45.4
MS20	54.84	MS45	45.61	MS70	51.74
MS21	44.96	MS46	43.29	MS71	53.76
MS22	50.14	MS47	43.44	MS72	73.49
MS23	60.25	MS48	42.6	MS73	83.7
MS24	45.48	MS49	46.09	MS74	43.1
MS25	52.52	MS50	53.53	MS75	42.49
MS76	45.65	MS101	53.26	MS126	45.12
MS77	53.04	MS102	50.35	MS127	43.88
MS78	54.64	MS103	60.89	MS128	45.02
MS79	67.87	MS104	59.96	MS129	46.9
MS80	64.1	MS105	46.72	MS130	54.6
MS81	59.19	MS106	47.93	MS131	43.77
MS82	45.33	MS107	41.7	MS132	91.04
MS83	44.66	MS108	61.74	MS133	59.07
MS84	43.46	MS109	90.4	MS134	44.67
MS85	56.5	MS110	57.47	MS135	59.7
MS86	50.94	MS111	62.52	MS136	81.31
MS87	31.61	MS112	53.82	MS137	70.2
MS88	43.42	MS113	53.78	MS138	64.51
MS89	38.4	MS114	51.79	MS139	86.28
MS90	67.22	MS115	59.49	MS140	91.4
MS91	44.04	MS116	53.08	MS141	59.05
MS92	91.4	MS117	50.75	MS142	49.35
MS93	51.08	MS118	52.38	MS143	38.11
MS94	60.9	MS119	49.4	MS144	33.07
MS95	33.68	MS120	59.1	MS145	57.17
MS96	43.47	MS121	53.55	MS146	41.21
MS97	55.28	MS122	47.91	MS147	56.87
MS98	50.42	MS123	54.7	MS148	28.24
MS99	68.38	MS124	47.81	MS149	51.69
MS100	66.57	MS125	45.41	MS150	47.49

Aflatoxins concentrations were quantified by using mReader Software by measuring the intensity of band developed on Reveal Q+ aflatoxin test strips. Detection limit for aflatoxins was 2–150 ppb

**Table 3:** Concentrations of aflatoxins contaminated maize samples as determined by thin layer chromatography

Sample no.	Concentration of aflatoxins (ppb)	Sample no.	Concentration of aflatoxins (ppb)	Sample no.	Concentration of aflatoxins (ppb)
MS1	43	MS26	56	MS51	35
MS2	36	MS27	51	MS52	44
MS3	51	MS28	34	MS53	36
MS4	44	MS29	37	MS54	44
MS5	39	MS30	52	MS55	65
MS6	45	MS31	51	MS56	52
MS7	57	MS32	62	MS57	49
MS8	57	MS33	36	MS58	52
MS9	35	MS34	47	MS59	78
MS10	48	MS35	48	MS60	76
MS11	52	MS36	61	MS61	81
MS12	48	MS37	44	MS62	85
MS13	55	MS38	31	MS63	62
MS14	36	MS39	51	MS64	83
MS15	75	MS40	46	MS65	54
MS16	48	MS41	42	MS66	65
MS17	59	MS42	43	MS67	43
MS18	57	MS43	65	MS68	31
MS19	37	MS44	39	MS69	35
MS20	55	MS45	51	MS70	52
MS21	45	MS46	44	MS71	54
MS22	52	MS47	35	MS72	74
MS23	63	MS48	43	MS73	81
MS24	46	MS49	47	MS74	43
MS25	55	MS50	51	MS75	31
MS76	45	MS101	51	MS126	46
MS77	53	MS102	52	MS127	44
MS78	51	MS103	61	MS128	45
MS79	68	MS104	48	MS129	48
MS80	64	MS105	45	MS130	51
MS81	59	MS106	42	MS131	44
MS82	56	MS107	42	MS132	81
MS83	48	MS108	62	MS133	52
MS84	37	MS109	91	MS134	41
MS85	51	MS110	58	MS135	60
MS86	51	MS111	57	MS136	79
MS87	30	MS112	59	MS137	75
MS88	41	MS113	56	MS138	61
MS89	32	MS114	52	MS139	87
MS90	64	MS115	60	MS140	82
MS91	47	MS116	54	MS141	60
MS92	86	MS117	51	MS142	51
MS93	54	MS118	53	MS143	35
MS94	62	MS119	47	MS144	32
MS95	34	MS120	60	MS145	51
MS96	38	MS121	57	MS146	42
MS97	56	MS122	48	MS147	51
MS98	52	MS123	54	MS148	20
MS99	65	MS124	42	MS149	58
MS100	69	MS125	41	MS150	48

Aflatoxins concentrations were quantified by comparing with the standards developed on thin layer chromatography. Detection limit for aflatoxins was 2–200 ppb

### Fungal mycoflora of different maize samples

**Fig. 1:**
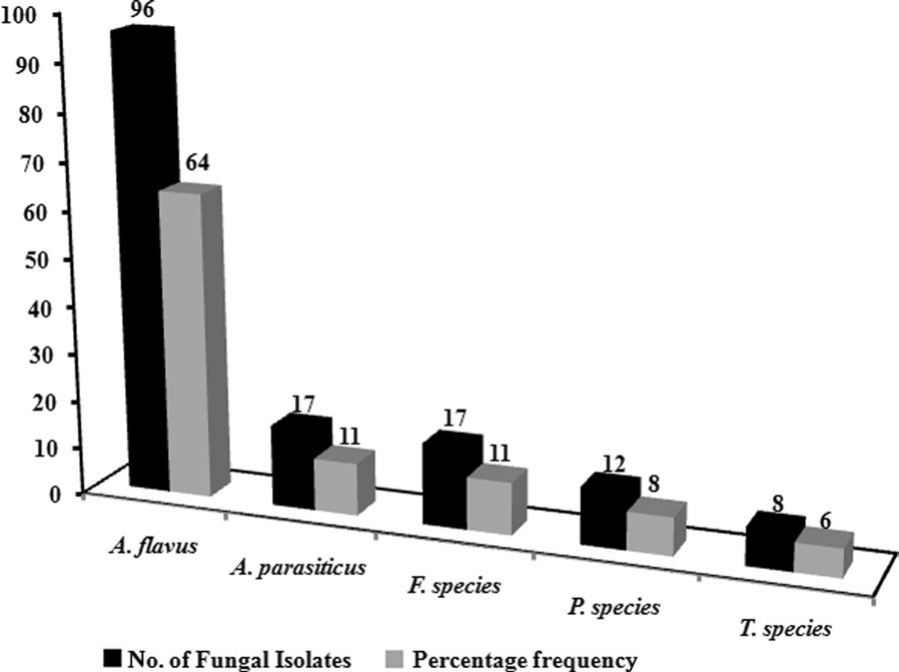
Distribution of fungal mycoflora among maize samples from Dilla region, Ethiopia. One ml of sample solution was aseptically spread on Potato Dextrose Agar and plates were incubated at 30 °C for 3–5 days. After incubation fungus were identified to genus and species level by referring standard protocol

The different load for fungal mycoflora of maize samples from Dilla region is highlighted in Fig. 1. Identification of fungal strain by standard protocol revealed that *Aspergillus* genus was predominant among maize samples which accounts for 75 % (113 samples). Among *Aspergillus* species, *A. flavus* accounts for 64 % (96 samples) followed by *A. parasiticus* with a frequency of 11 % (17 samples). Apart from *Aspergillus* fungi, *Fusarium* spp, *Penicillium* spp and *Trichoderma* spp were also isolated among various maize samples studied. *Fusarium* spp contamination contributed 11 % (17 samples) while, *Penicillium* spp and *Trichoderma* spp shares 8 % (12 samples) and 6 % (8 samples) respectively (Fig. 1).

## Discussion

Aflatoxins contamination of crops possesses a serious threat to human and animal health as well as consider as danger in trade market (Bennett and Klich 2003). Among various mycotoxins produced by fungus, aflatoxins has distinct relation with maize requires serious concerns in decontamination of toxicity in many agricultural commodities (Trung et al. 2008). Even though maize in one of the most important crop than wheat and teff in Ethiopia, maize are not well studied for the toxicity generated by aflatoxins. Aflatoxins are reported to be prevalent through the west and east Africa. Some of the previous studies reported that 90 % of east African maize samples showed the evidence of high level of aflatoxins, and some parts of West Africa the exposure of aflatoxins is as high as 99 % (Doko et al. 1995; Shephard 2004; Rodrigues et al. 2011). In comparison to east and West Africa, Ethiopia has a serious problem with aflatoxins though the exact levels of exposures are uncertain due to lack of data or testing (Bernard et al. 2008). In the proposed study, all the samples come from the regions within the temperature ranges from 20 to 31 °C (Alene et al. 2000). Earlier studies demonstrated that higher temperature supports the growth of *Aspergillus* species (Chauhan et al. 2008). In addition to the above factor, farmers are not aware of handling of crops and storage in this part of country. They did not follow the standards for the processing of maize samples. Therefore possibilities of contamination of food commodities employed for human consumption in this region cannot be ruled out. The results of our study confirmed that all the samples utilized in this study are at a risk of contamination of aflatoxins. As shown in Tables 2 and 3, more than 50 % of samples possess aflatoxin concentration more than 50 ppb. In addition to this, the average mean concentration of aflatoxin was resulted as 53 and 52.1 ppb as determined by immunochromatograhpic assay and thin layer chromatography respectively.

Aflatoxins not only support severe health risk but also favours significant economic lost to farmers whether their crops must be rejected or accepted for buyers. For example in Kenya, two World Food Program of the United Nation purchased maize samples were confiscated and destroyed because of the lack of acceptable levels of aflatoxins in the purchased crops (Hassan et al. 1998). This is of particular concerns to smallholder farmers as aflatoxins toxicity primarily emerge out where there is high moisture content and high temperatures which is supported by inadequate storage structures. The place visited in this study fulfils all of these criteria and was confirmed by our study that aflatoxins contamination is serious challenge to smallholder farmers especially in this part of country.

Previous studies from neighbouring countries of Ethiopia like Kenya, Somalia, Uganda and Sudan demonstrate that *A. flavus* and *A. parasiticus* can invade maize seed in the field before harvest, during post harvest, drying and curing as well as during storage and transportation. Since, spores of both the species can survive for a long period of time in air and can get disseminated over a long period of distance from one place to another (Bhat et al. 1997; Gao et al. 2007). Since, Dilla town is located on the Addis Ababa-Nairobi international highway, there is potential of dissemination of spores from Kenya to Ethiopian commercial outlets as well as in maize fields. Our data confirmed the presence of *Aspergillus* as dominant fungal mycoflora among all which accounts for 75 % of samples followed by *Fusarium* (11 %), *Penicillium* (8 %) and *Trichoderma* (6 %) (Fig. 1).

The prevalence of contamination of maize sample in this study by aflatoxins is consistent with previous reports from this country (Abera and Admssu 1988; Habtamu and Kelbessa 2001) and in other countries with same climatic conditions (Shephard 2004). However within Ethiopia, a national standard has yet to be set the regulatory acceptable levels of aflatoxins. Therefore it is difficult to say that really the maize samples are acceptable or rejectable for human consumption base on our study. But in comparison to regulatory levels of aflatoxins with other countries the concentration of aflatoxins found in the samples of this study are quite higher when compared with their respective setting limits. Based on that we can recommend that maize samples analyzed in these findings correspond to heavier toxicity of aflatoxins and requires setting of safety levels for mycotoxins by respective bodies of the countries immediately.

Humans are exposed to aflatoxins mostly by consuming contaminated foods containing fungal metabolites at threshold levels. Most of the developing countries in Africa, risk of aflatoxins contamination have been companion with increase risk of hepatocellular carcinoma and esophageal cancer respectively (CAST 2003; Murugavel et al. 2007). Although there is no direct evidence still available that demonstrate that aflatoxins affected food consumption leads to cancer in Ethiopia. Therefore findings of these reports emphasize that the presence of aflatoxins at high concentration in maize samples may related to serious public health concerns and assured that fungal toxicity is a major problem in this country. Since no agricultural commodities are not directly prone to mycotoxins contamination, results of this work will guide the identification of various factors responsible for contamination and the areas where control measures requires serious intervention. Implementation of national prevention and control strategies like proper pre-harvest and pro-harvest treatment of infected maize and standard storage facilities are required to reduce the risk of aflatoxin contamination by fungi. In addition to this more studies are required from different parts of Ethiopia to generate data for Ethiopian government to work on policy making decision strategy. More importantly there is a need to find out whether aflatoxins are dominant among mycotoxins in maize or chances of contamination of other mycotoxins other than aflatoxins are prevalent. Since in our study, 25 % of mycoflora was not *Aspergillus* but governed by other fungal species like *Fusarium*, *Penicillium*, *Trichoderma* that are known to produce different kinds of mycotoxins.

## Conclusions

The maize samples collected from Gedeo zone, Ethiopia were contaminated with aflatoxins. Due to the levels of aflatoxins observed in this work posses a potential threat to the agricultural industry and require urgent intervention. It is important to undertake control strategies and to distinguish the maize samples whether suitable for human consumptions and animal feed or not. These results emphasize the need for future research to reduce the occurrence of aflatoxins contamination in Ethiopian maize.
